# Supplementary material for: Bugs on Drugs: A Drosophila melanogaster Gut Model to Study In Vivo Antibiotic Tolerance of E. coli
Source: Microorganisms. 2022 Jan 7;10(1):119. doi: 10.3390/microorganisms10010119 (PMC8780219; doi:10.3390/microorganisms10010119)
Supplement: Supplementary file 1 [file microorganisms-10-00119-s001.zip › microorganisms-1512349-supplementary.pdf]

microorganisms

1. Supplementary files

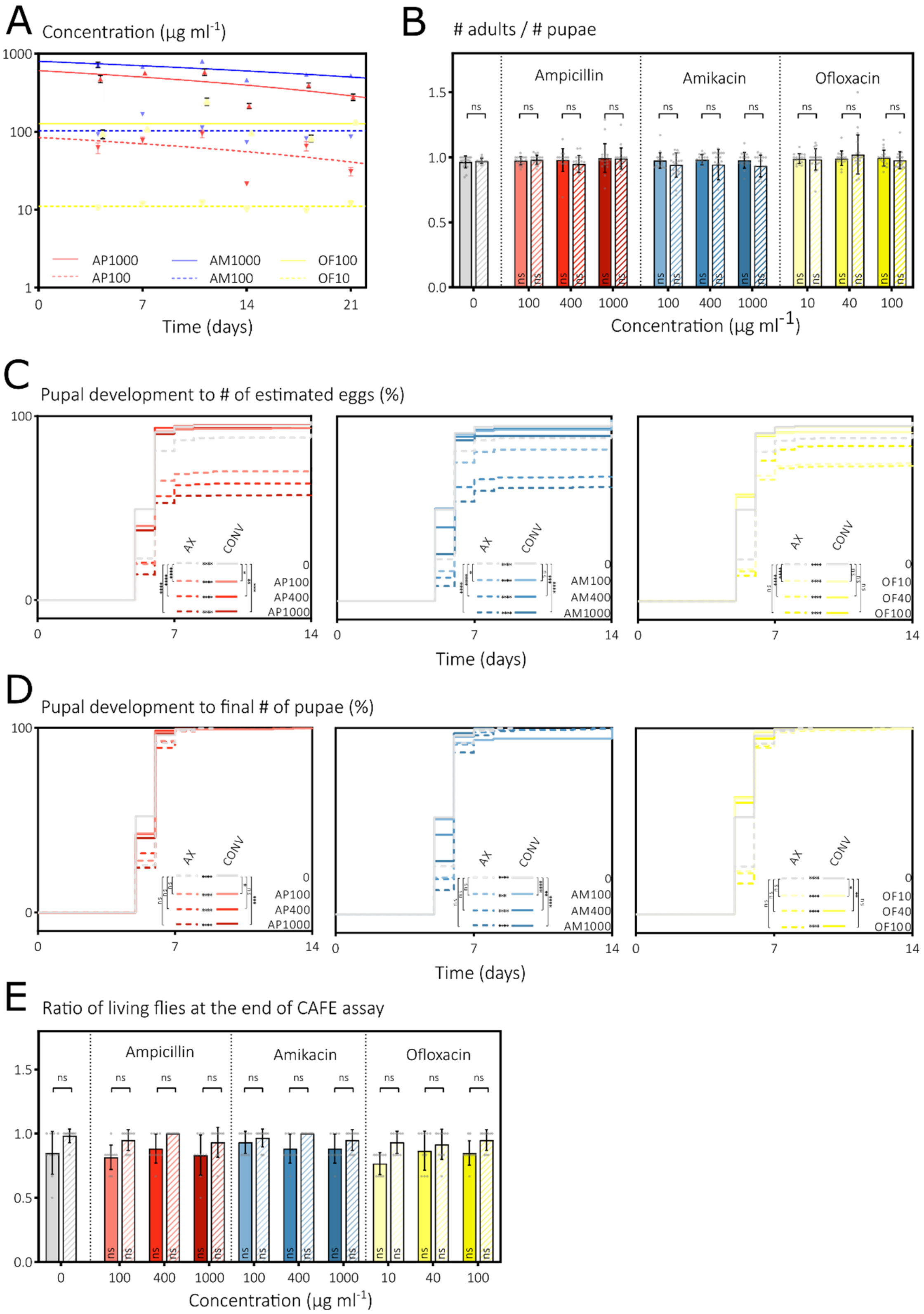

Figure S1. Direct effects of antibiotics on *Drosophila melanogaster* are limited to lethality in the axenic, larval stage. **(A)** Antibiotics are reasonably stable over the course of 14 days as assessed by a bioassay (see methods). Concentrations decreased significantly when starting with AP100, AP1000 and AM1000 (at rates of -2.1, -15.2 and -14.2 µg ml^{-1} day^{-1} respectively) in the food while for the others, no decrease was observed based on AIC comparing a linear fit to a horizontal line (means ± sems with n= 6 per group). **(B)** Antibiotics have no lethal effect on the development to adults once larvae reach the pupal stage. As

in Fig 1A, conventional eggs (CONV, full bars) or axenic eggs (AX, dashed bars) were deposited on the surface of nutrient agar containing either no antibiotic (gray) or AP (red), AM (blue) or OF (yellow) at different concentrations (shades). The ratio of the cumulative number of adults by day 14 to the cumulative number of pupae over the course of the experiment was plotted (means ± stdevs with n=± 800 per group across 20 vials as gray points). A linear mixed model (lme) with post hoc tests for to the untreated control (Dunnett per AX and CONV groups, in bar) and between AX-CONV per treatment (Tukey, above bar) resulted in no significant effects (ns). **(C)** The AX group develops more slowly compared to CONV group, and even more so when receiving antibiotic treatment. Developmental dynamics are plotted as inverse survival plots and Cox mixed survival models with post hoc test analysed differences between AX and CONV groups (Tukey, between legenda lines) and between untreated and treated groups (Dunnet, brackets in legenda) (ns, non-significant; *, $P < 0.05$; **; $P < 0.01$; ***, $P < 0.001$; ****, $P < 0.0001$). **(D)** As in (C) but here, uncensored data are plotted (i.e., re-analysing all datasets assuming that all eventual pupae equals total number initial pupae) thereby disregarding any pupal lethality. This way, any differences between untreated and treated AX groups in (C) disappear. **(E)** During capillary feeding, there is no significant effect of either microbial status (AX or CONV) or antibiotic treatment on the survivorship of adult flies over the timespan of 4 days as apparent from post hoc tests on a zero-inflated model for significant differences between AX-CONV per treatment (Tukey) and with the untreated control (Dunnett). Numbers and setups as in Figure 1B.

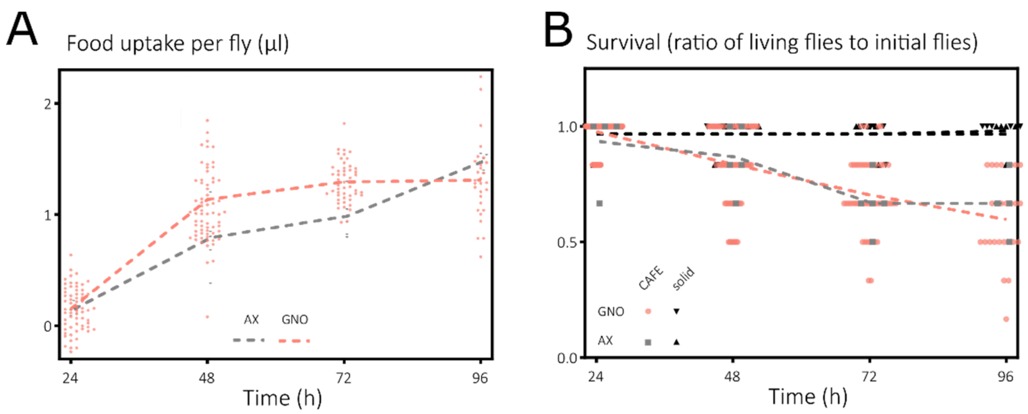

Figure S2. Gnotobiotic flies associated with *E. coli* display similar feeding and mortality compared to an axenic control group. **(A)** When associated with *E. coli,* gnotobiotic *D. melanogaster* flies (GNO in red, 9-10dpe) show a highly similar feeding pattern in the CAFE assay than its axenic counterparts of the same age (AX, in gray) or of younger age (2-3dp, see Figure 1B). Food uptake was normalized per fly (means, n=≥29 vials for GNO across ≥2 runs and n = 5 for AX). **(B)** Both GNO flies (red points) and AX flies (gray squares) suffer from high mortality during the CAFE assay. In contrast, rearing on solid food has only limited effect on the survivorship of GNO flies (black downward triangles) or AX flies (black upward triangles). Survival per vial was computed per day as the ratio of living individuals to its initial (means, n=≥29 vials for GNO on CAFE across ≥2 runs and n = 5 for the other groups). Data for the same experiment as depicted in Figure 2A.

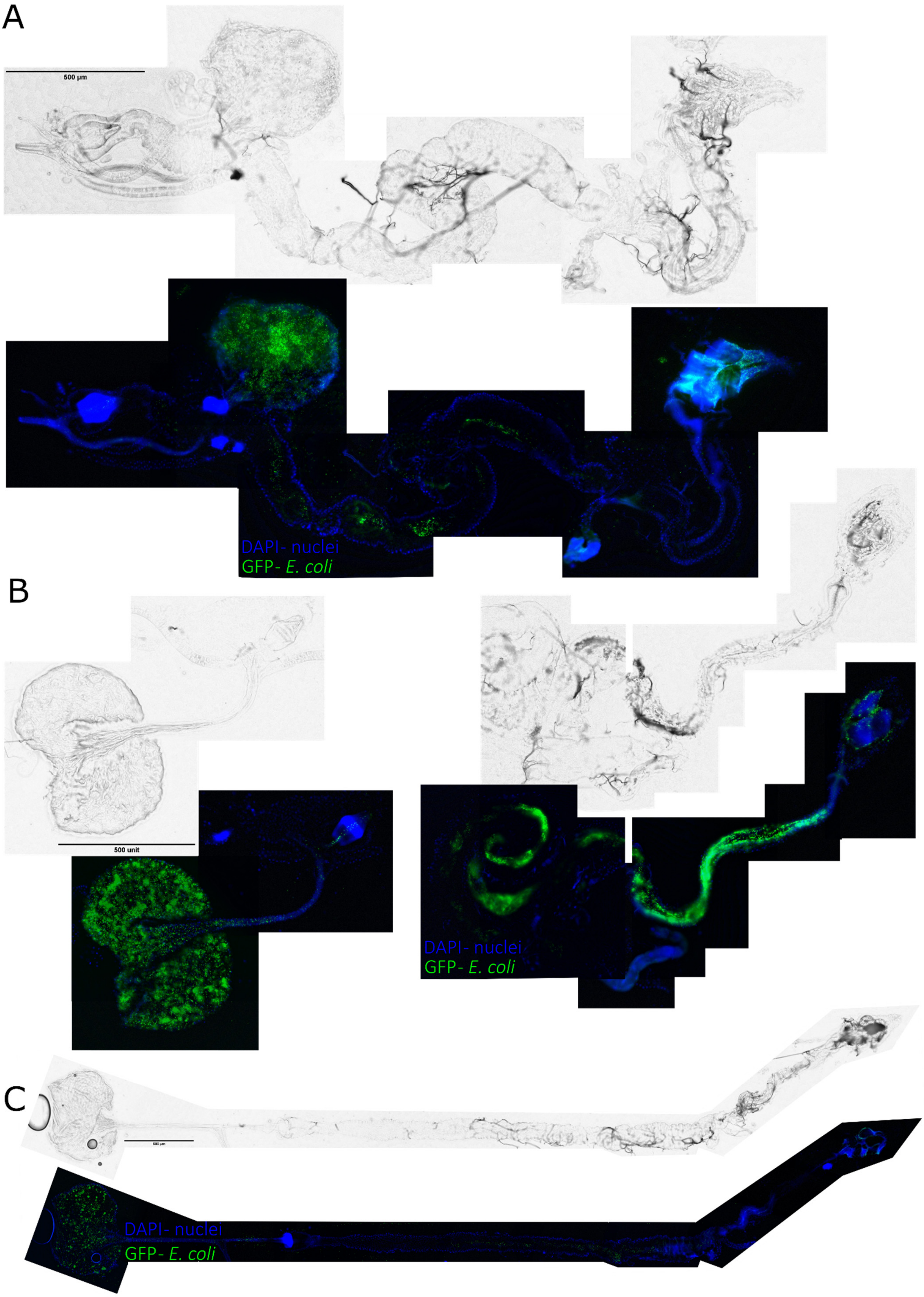
A
500 µm
DAPI- nuclei
GFP- E. coli
B
500 unit
DAPI- nuclei
GFP- E. coli
C
500 µm
DAPI- nuclei
GFP- E. coli

Figure S3. *E. coli* is taken up orally by axenic *D. melanogaster* flies and is specifically enriched in the crop. **(A)** and **(B)** are both dissected crops of AX flies directly after the association-by-feeding setup when fluorescent bacteria can be found throughout the gut. **(C)** shows an additional representative micrograph of a dissected gut of a fly that has been associated with *E. coli* through feeding and additionally has been reared on sterile, solid fly food for another week. As such, (C) is similar to Figures 2B-D in the main text. While bacteria can also be found throughout the gut at this timepoint (9-10 dpe) and under these conditions, *E. coli* seems especially to be enriched in the crop of the fly. Individual microscopy images were recorded at 20x magnification at a single plane in *z* dimension and adjusted to have an equal brightness when stitched together (see methods).

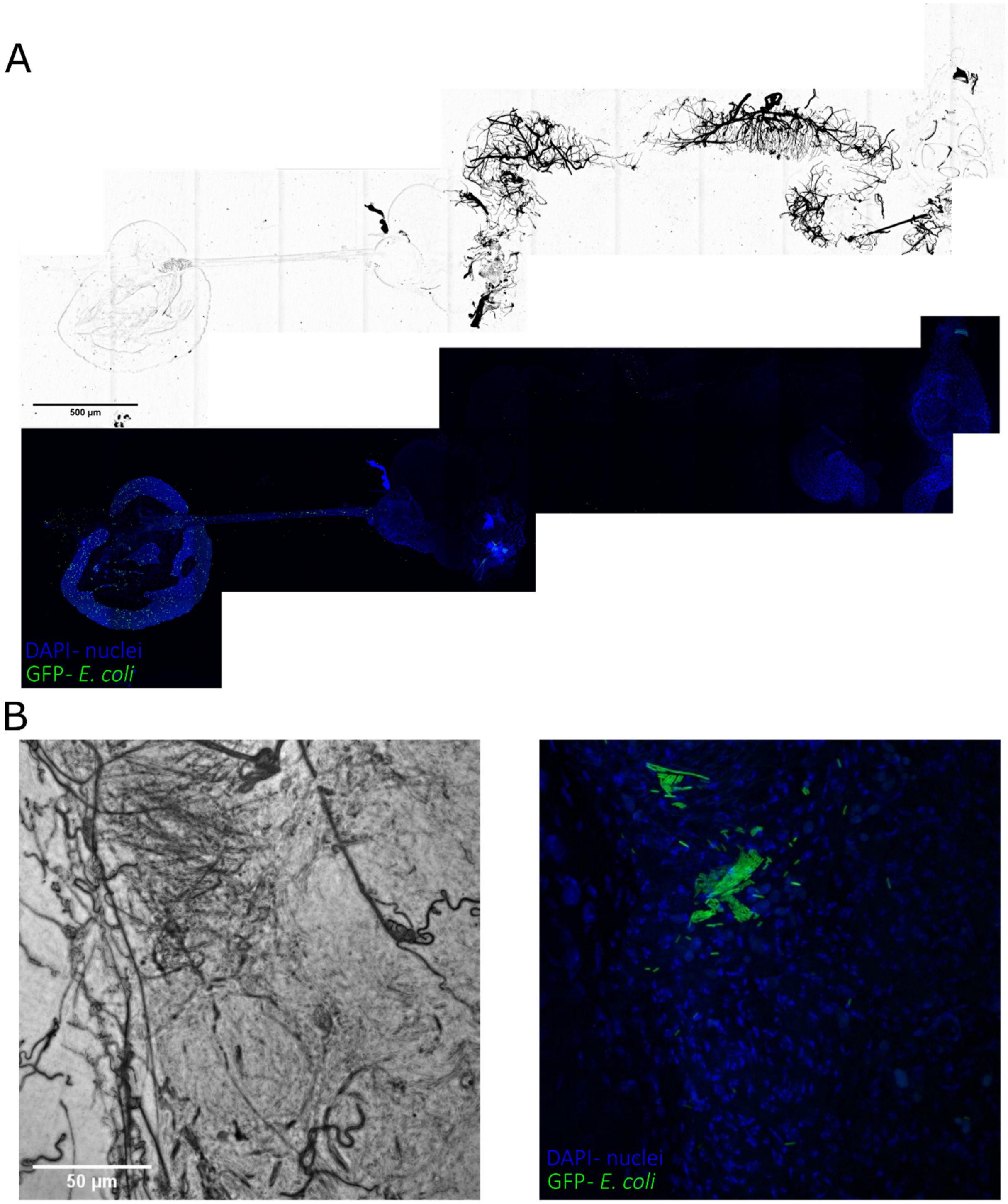

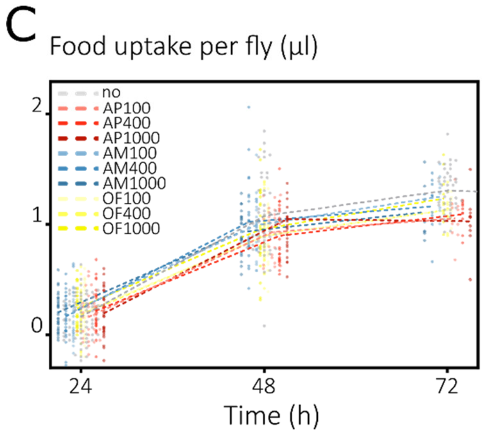

D

	Uptake (µl)	Dose in fly (g kg^{-1} day^{-1})	Human dose (g kg^{-1} day^{-1})
AP100	0.93-1.06	0.018-0.023	0.011
AM100	0.99-1.25	0.186-0.211	0.169
OF10	0.90-1.14	0.199-0.251	0.015

Figure S4. Gnotobiotic flies maintained under the CAFE assay show microcolonies of *E. coli* in the crop and show a regular feeding rate leading to an antibiotic dosage similar than in human. **(A)** Under the CAFE assay, *E. coli* remains located specifically in the crop of *D. melanogaster* flies (12-13 dpe). Confocal images of dissected crops at a 20x magnification were automatically stitched together while recording and afterwards, *z* stack images were projected onto one plane along with other adjustments for visual representation (see methods). The top shows the widefield image with scalebar, the bottom combines the DAPI (*Drosophila* nuclei) and GFP (*E. coli* cells) channels of the top point of view. **(B)** In the crop, *E. coli* cells regularly occur in a patched, microcolony alike fashion indicative of *in vivo* proliferation or biofilm growth. A crop section was imaged at 63x magnification using confocal microscopy, with similar adjustments for visual representation as in (A). **(C)** During the CAFE assay when *D. melanogaster* flies associated with the SX43 wild-type *E. coli* strain, are offered liquid food containing AP, AM, or OF at different concentrations, flies display a similar feeding pattern as before (Figures 1B and S2A). Food uptake was normalized per fly (means, n=≥10 vials across ≥2 runs). **(D)** This food uptake results, even at the lowest antibiotic concentration, in a dosage that exceeds the suggested dosage in human for severe infections. The uptake is the minimum-maximum uptake at 48h and 72h combined and the weight of a male fly is approximated by 500 µg [96]. The human dosage was obtained from the Merck MSD [97].
